# Experimental investigation of heat transfer coefficient in pool boiling of hybrid nanofluid over grooved surfaces

**DOI:** 10.1038/s41598-025-27380-4

**Published:** 2025-11-28

**Authors:** Amir Vasei Moghadam, Hamid Reza Goshayeshi, Vahid Nejati

**Affiliations:** Department of Mechanical Engineering, Ma.C., Islamic Azad University, Mashhad, Iran

**Keywords:** Heat transfer coefficient, Grooved surfaces, Heat flux, Hybrid nanofluid, Pool boiling, Engineering, Materials science, Mathematics and computing, Nanoscience and technology, Physics

## Abstract

Heat transfer in pool boiling is known as a complex and critical process in thermal systems. Considering the escalating need for higher efficiencies, using nanofluids as an efficient fluid in addition to modifications to surface geometry- particularly grooved surfaces- has been identified as an effective strategy for enhancing the heat transfer coefficient. This research seeks to explore the experimental effects of a hybrid graphene oxide-iron oxide/water nanofluid combined with grooved surfaces on the convective heat transfer coefficient during pool boiling. Tests were performed using a hybrid nanofluid with a 0.05% volumetric concentration on surfaces featuring diverse groove configurations. The findings demonstrated that the use of hybrid graphene oxide-iron oxide/water nanofluid alongside grooved surfaces substantially enhances the heat transfer coefficient. This enhancement stems from the synergistic influence of nanoparticles and surface geometry on the boiling process, coupled with increased turbulence in the liquid boundary layer. Among the tested configurations, the circular surface in the hybrid nanofluid exhibited the highest heat transfer coefficient improvement. Compared to a smooth surface with deionized water, the heat transfer coefficient increased by 67%. This study offers promising insights for advancing heat transfer technologies and designing advanced cooling systems. It also introduces the use of hybrid nanofluids along with engineered surfaces as a new approach to optimizing thermal processes.

## Introduction

Pool boiling is a widely employed heat transfer mechanism in energy systems, electronic cooling, and nuclear reactors due to its ability to achieve high heat transfer rates and sustain large heat fluxes. Despite its advantages, limitations such as the critical heat flux (CHF) and the heat transfer coefficient (HTC) restrict its effectiveness. Several strategies have been proposed to improve boiling performance, including the addition of nanoparticles to base fluids, surface modification to expand the heater area, and external perturbations such as vibrations^1^.

The introduction of nanofluids, pioneered by Choi^2^, has attracted significant attention in recent decades. Nanoparticles, owing to their superior thermal conductivity compared to conventional fluids, can enhance boiling performance by modifying thermal and interfacial properties. Studies have shown that nanoparticle concentration, size, and material type strongly influence HTC and CHF. Shi et al.^3^ demonstrated that Al_2_O_3_/water nanofluid improves HTC due to higher conductivity and reduced surface tension, while Du et al.^4^ reported that Fe_3_O_4_/water nanofluid improves both HTC and CHF through nanoparticle deposition and surface morphology changes. Similarly, Vafaei^5^ observed that higher nanoparticle concentrations may reduce HTC at high fluxes because of agglomeration effects.

Other studies highlight how nanoparticle type and concentration alter performance differently. For example, Umesh et al.^6^ studied CuO/pentane and observed enhancements with fine fin surfaces but reductions with coarse fins, attributed to capillary effects and bubble detachment resistance. Graphene-based nanofluids have also shown promise: Khan et al.^7^ and Singh et al.^8^ reported significant improvements in HTC and CHF with GO- and rGO-based fluids, particularly at optimal concentrations. Carbon nanotube nanofluids^9^ and silica-based systems^10,11^ further confirmed the role of nanoparticle-induced wettability and porous layer formation in enhancing CHF.

Nevertheless, contradictory findings also exist. For instance, Vassallo et al.^12^ reported negligible improvement with SiO_2_/water nanofluids, while Lee et al.^13^ highlighted enhanced CHF but no significant HTC gain in TiO_2_- and Al_2_O_3_-based systems. Such inconsistencies underscore the importance of nanoparticle type, morphology, and experimental conditions.

Building upon single nanofluid systems, hybrid nanofluids have emerged as a promising alternative to combine the advantages of multiple nanoparticles. Several studies demonstrated superior HTC and CHF enhancements compared to single-particle nanofluids. For example, Sharma et al.^14^ showed that Ag/ZnO hybrids improved both HTC and CHF, while Kamel et al.^15^ reported strong performance from an Al_2_O_3_/CeO_2_ blend. Mehralizadeh et al.^16^ observed significant HTC enhancement with TiO_2_/SiO_2_ hybrids compared to single nanofluids. Yagnem et al.^17^ studied CuO–Al_2_O_3_/water and noted performance improvements up to a critical concentration, beyond which deterioration occurred. Ma et al.^18^ tested graphene–silver/water hybrids and identified an optimal CHF enhancement at 0.001 wt%. Similarly, Aizzat et al.^19^ reported varying boiling behavior for Al_2_O_3_/SiO_2_ hybrids, with performance depending on concentration and nanoparticle ratio. Gupta et al.^20^ recorded peak increases of over 200% in HTC with Cu–ZnO hybrids, while Saalim et al.^21^ showed that Al_2_O_3_–CuO hybrids outperform single nanofluids in both HTC and CHF. Collectively, these findings suggest that hybrid nanofluids can exploit synergistic effects, though optimal concentrations and long-term stability remain challenging.

Parallel to nanofluid research, surface modification has been extensively studied to enhance boiling performance. Engineered surfaces such as roughened, porous, grooved, and finned geometries improve boiling by increasing nucleation site density, promoting bubble detachment, and enhancing liquid rewetting. Das et al.^22,23^ showed that perforated and grooved surfaces outperform smooth ones due to increased nucleation sites and expanded thermal contact. Narayan et al.^24^ introduced a roughness parameter (φ) that determines whether nanoparticles block or activate nucleation sites, depending on concentration and size. Pastuszko et al.^25^ observed that micro-finned surfaces coated with porous copper significantly enhanced HTC. Other modifications, such as inclined^26^, hooked^27^, and V-shaped porous surfaces^28^, have also been shown to improve boiling efficiency.

Grooved surfaces in particular have received considerable attention. Kumar et al.^29^ attributed HTC improvements on grooved surfaces to enhanced bubble dynamics, Gao et al.^30^ highlighted the role of micro-pores, and Falsetti et al.^31,32^ reported significant CHF and HTC increases with Novec-based fluids on grooved and roughened copper surfaces. Rashid et al.^33^ further demonstrated the complex interactions between groove geometry, fluid type, and boiling performance, where circular grooves significantly outperformed rectangular and triangular ones when using nanofluids.

Overall, the literature indicates that both fluid modification through nanoparticles and surface modification through geometric structuring can substantially improve pool boiling. However, studies that combine hybrid nanofluids with engineered surfaces remain limited. While single nanofluids or surface modifications alone can enhance HTC and CHF, their simultaneous application may yield synergistic benefits. In particular, graphene oxide (GO) and iron oxide (Fe_3_O_4_) hybrid nanofluids offer complementary advantages: GO provides high thermal conductivity and enhanced wettability, while Fe_3_O_4_ facilitates nucleation and modifies surface morphology.

The present work is confined to the nucleate boiling regime with emphasis on the heat transfer coefficient (HTC). Critical heat flux (CHF) was beyond the scope of this investigation and is left for future research. This study presents the first experimental investigation of a GO–Fe_3_O_4_/water hybrid nanofluid at 0.05% vol concentration on grooved surfaces under pool boiling conditions. The objective is to clarify the combined effects of hybrid nanofluids and surface geometry on HTC enhancement, benchmark the findings against existing studies, and provide insights into the design of advanced thermal management systems for high heat flux applications.

## Material, experimental apparatus, boiling surfaces

### Preparation of hybrid nanofluid

**Fig. 1:**
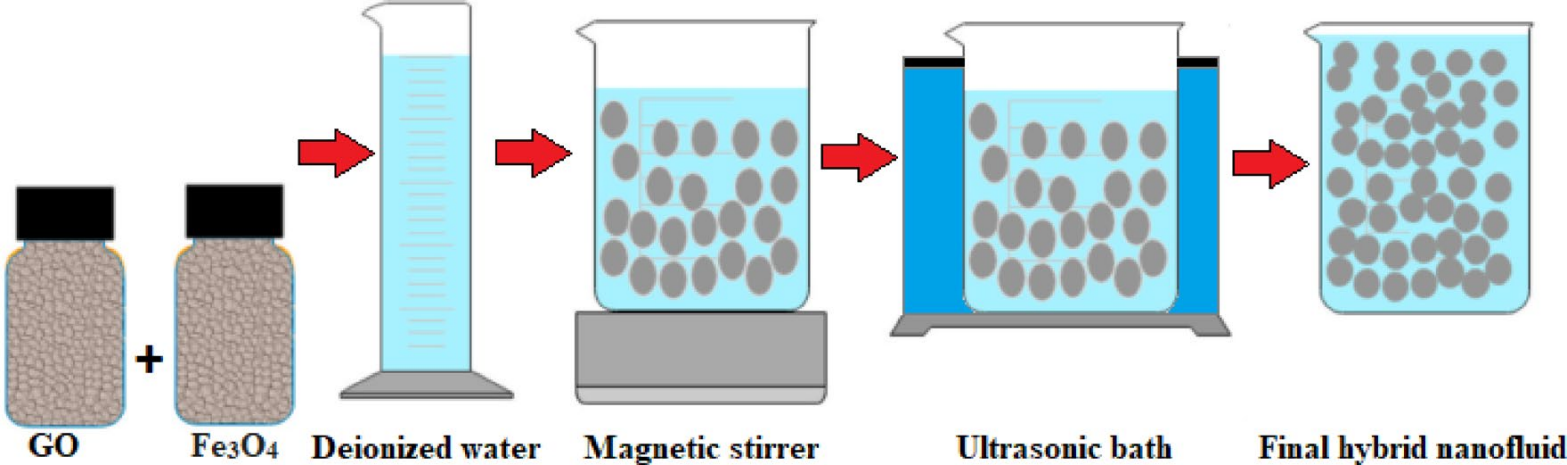
Schematic of hybrid nanofluid preparation.

**Fig. 2:**
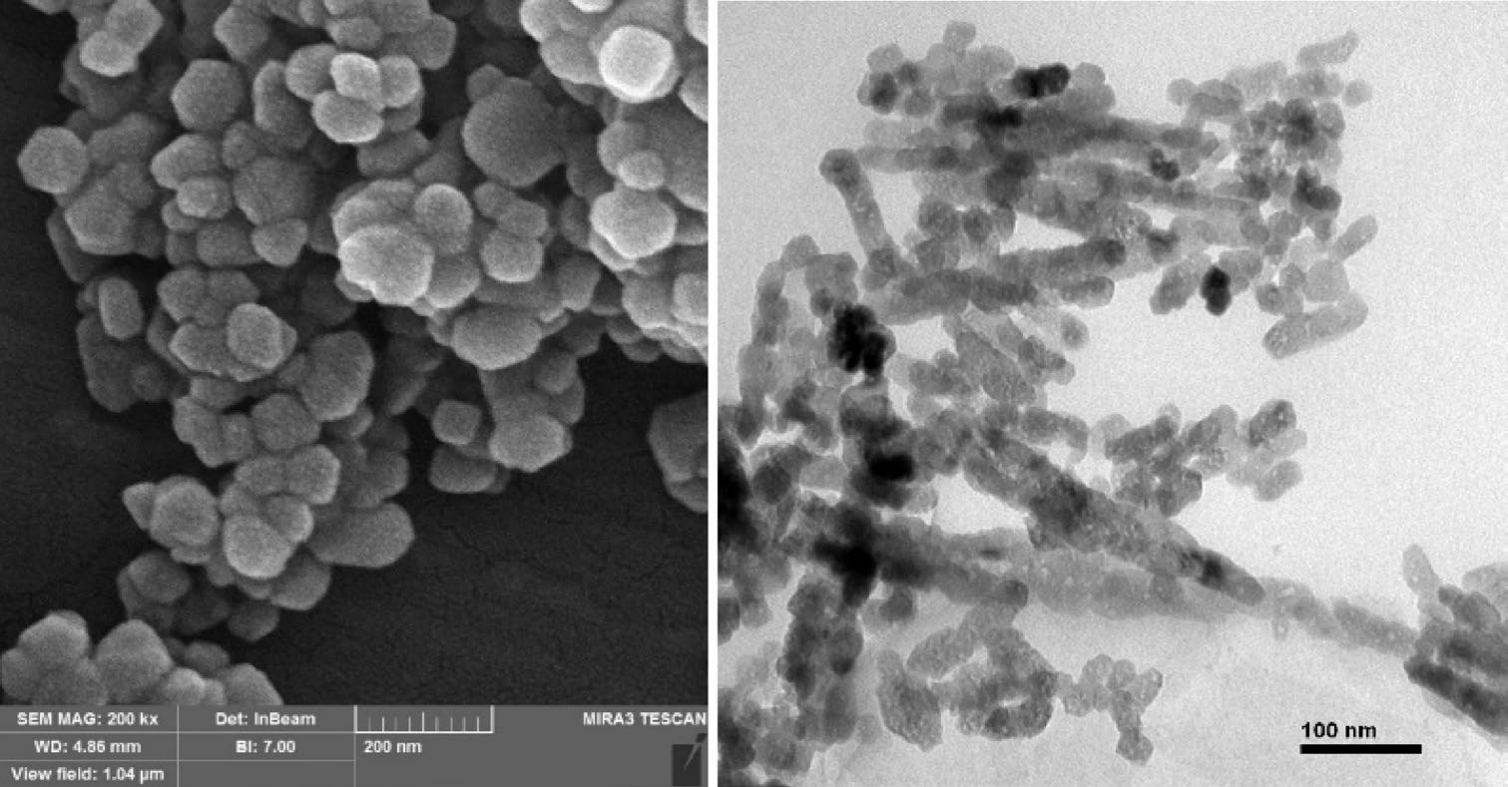
TEM and SEM images of iron oxide nanoparticle.

**Fig. 3:**
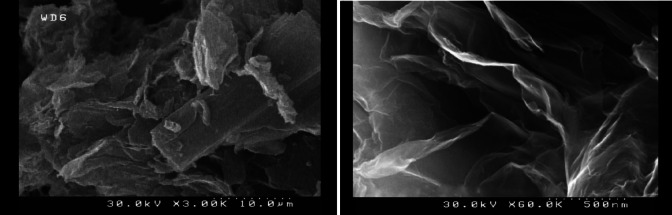
TEM and SEM images of graphene oxide nanoparticle.

To prepare a hybrid nanofluid comprising graphene oxide and iron oxide dispersed in water with a 50:50 ratio at a volumetric concentration of 0.05% based on Eq. (1), first 1.3 g of iron oxide and 0.9 g of graphene oxide are poured into 1000 ml of deionized water. This mixture was then blended using a magnetic stirrer for a duration of 90 min and subsequently placed in an ultrasonic bath for 120 min to stabilize the hybrid nanofluid, as shown in Fig. 1. Images from Transmission Electron Microscopy (TEM) and Scanning Electron Microscopy (SEM) showing the nanoparticles involved in this experiment are presented in Figs. 2 and 3, respectively. The general specifications of these nanoparticles are summarized in Table 1.

1 $$\upphi { } = \frac{{{\text{V}}_{{{\text{nanoparticles}}}} }}{{{\text{V}}_{{{\text{total}}}} }} \times 100$$

**Table 1:** Specification of the nanoparticles used in the experiments.

Property	Graphene oxide	Iron oxide
Chemical formula	GO	Fe_3_O_4_
Particle size	3.4–7 nm	20–30 nm
Appearance	Black powder	Black powder
Purity	≥ 99%	≥ 98%
Density	3600 kg m^−3^	5200 kg m^−3^
Thermal conductivity	3000 Wm^−1^ K^−1^	17.6 Wm^−1^ K^−1^

To evaluate the thermal conductivity coefficient of the hybrid nanofluid, the KD2 Pro thermal analyzer was employed, and a value of 0.891 Wm^−1^ K^−1^ was obtained at the concentration of 0.05 vol%. The density of the prepared hybrid nanofluid was determined using Eq. (2), giving 2698.5 kg m^−3^.

2 $$\uprho_{{{\text{hnf}}}} = \upphi_{{{\text{np1}}}} \uprho_{{{\text{np1}}}} + \upphi_{{{\text{np2}}}} \uprho_{{{\text{np2}}}} + \left[ {{1} - \upphi_{{{\text{np1}}}} - \upphi_{{{\text{np2}}}} } \right] \times \uprho_{{{\text{bf}}}}$$

**Fig. 4:**
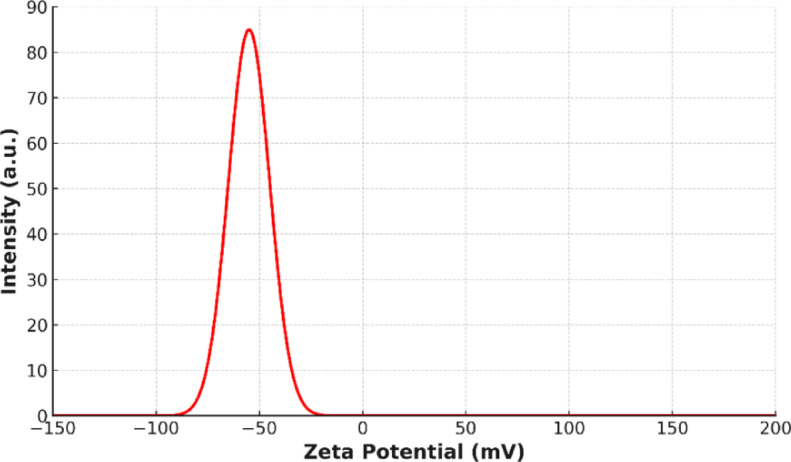
Absolute Zeta potential of 0.05 vol% hybrid nanofluid.

**Fig. 5:**
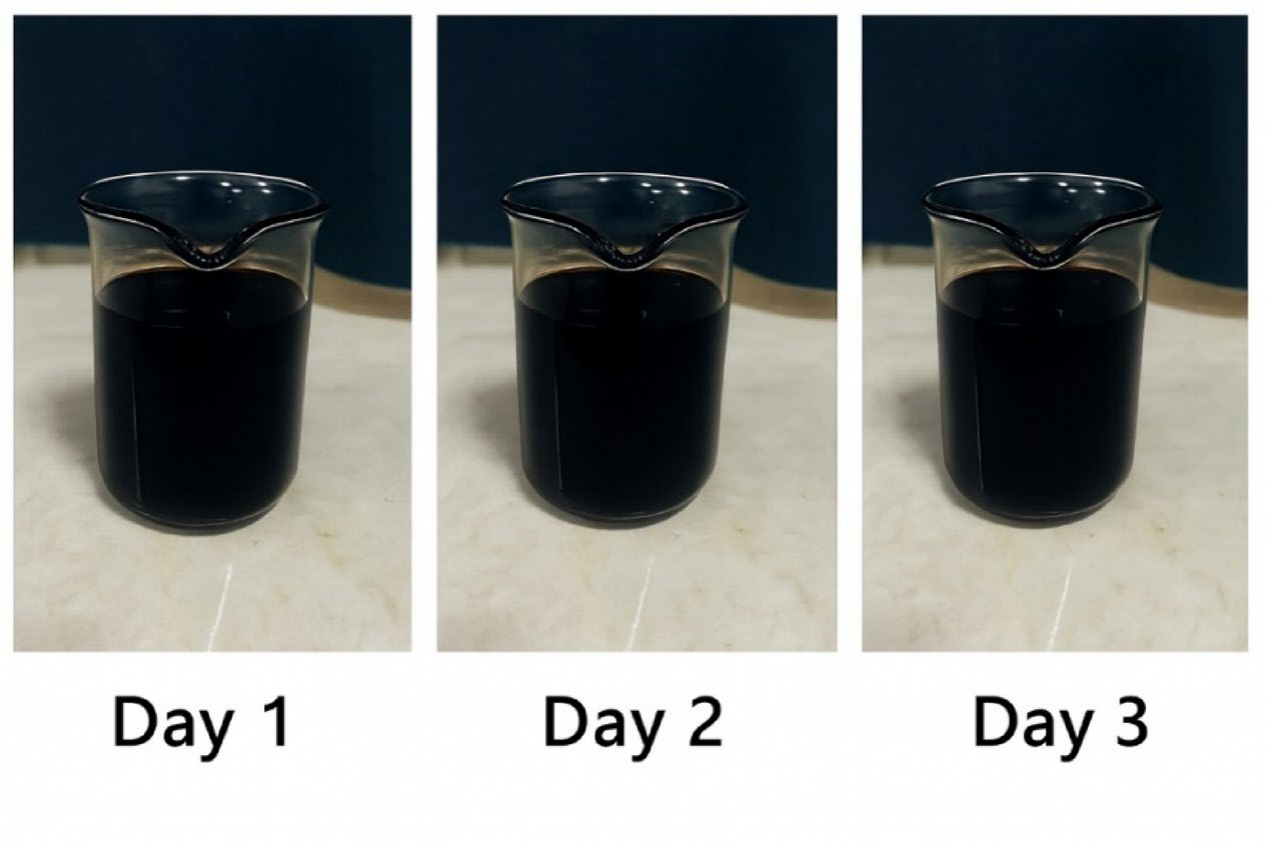
Stability of hybrid nanofluid over 3 days (Day 1, Day 2, Day 3).

In order to assess the stability of the prepared suspension, zeta potential measurements were carried out. As illustrated in Fig. 4, the hybrid nanofluid exhibited a peak zeta potential value of approximately − 55 mV, which is significantly higher than the ± 30 mV threshold commonly accepted as an indicator of excellent colloidal stability^34^. Furthermore, a photographic image of the nanofluid sample after 3 days Fig. 5 shows no visible sedimentation or agglomeration, further confirming the long-term stability of the prepared hybrid nanofluid.

**Fig. 6:**
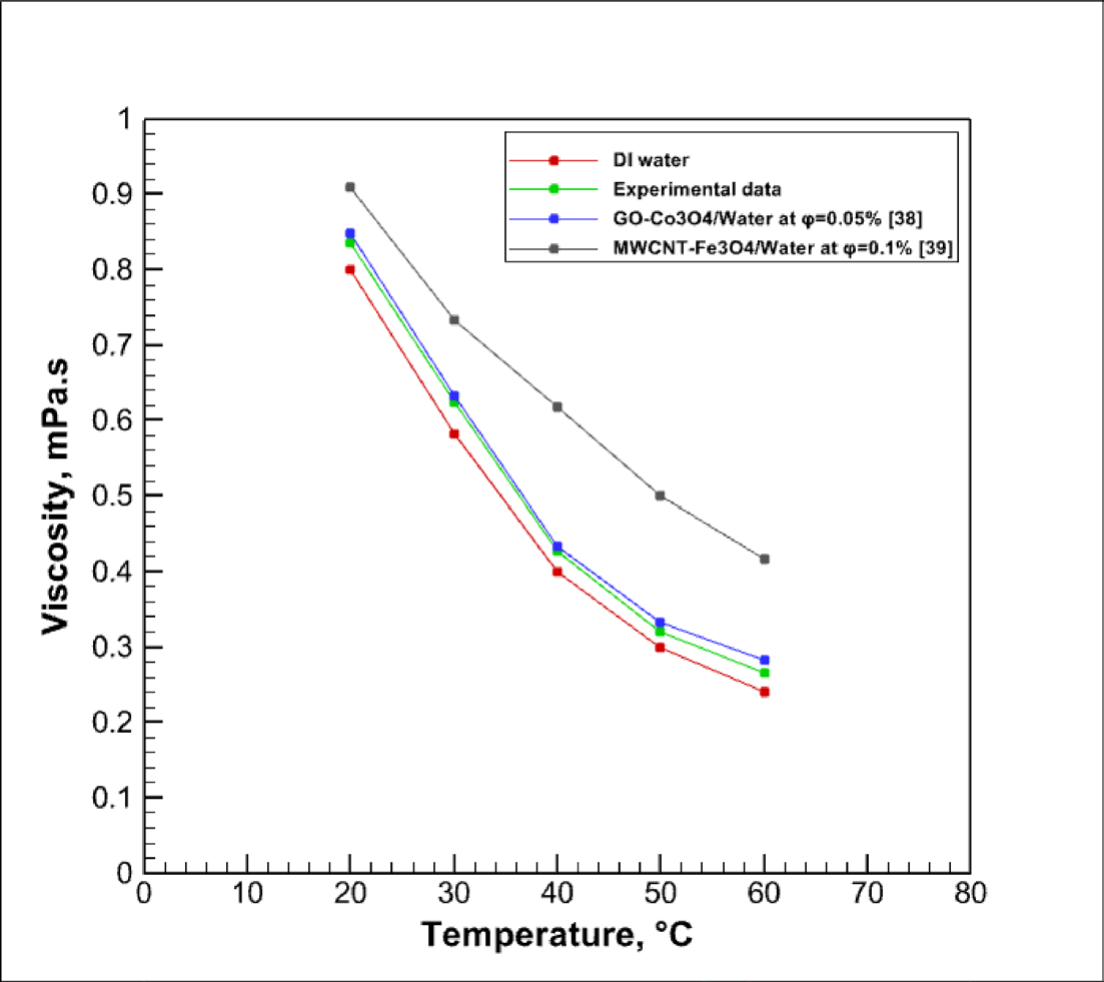
Experimental data of viscosity variation with temperature.

The viscosity of the hybrid nanofluid was measured experimentally using an NDJ-8S digital rotational viscometer at various temperatures to examine its temperature-dependent behavior. As expected, the viscosity decreases with increasing temperature, while it slightly increases with nanoparticle concentration. Figure 6 presents the experimentally measured viscosity of the hybrid nanofluid as a function of temperature, along with a comparison with previously published data^35,36^. A good agreement between the present data and literature results confirms the accuracy and reliability of the current measurements.

### Test apparatus

**Fig. 7:**
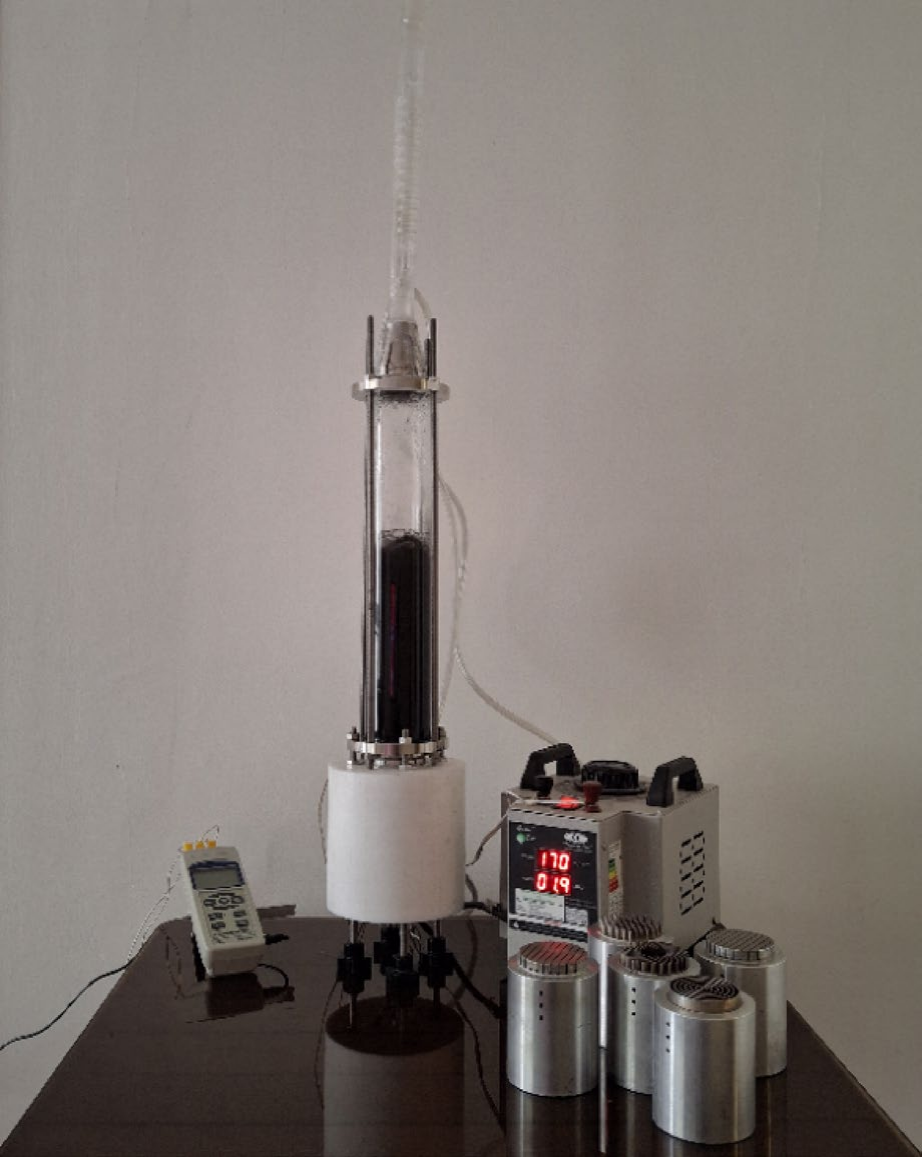
Actual image of the test apparatus.

**Fig. 8:**
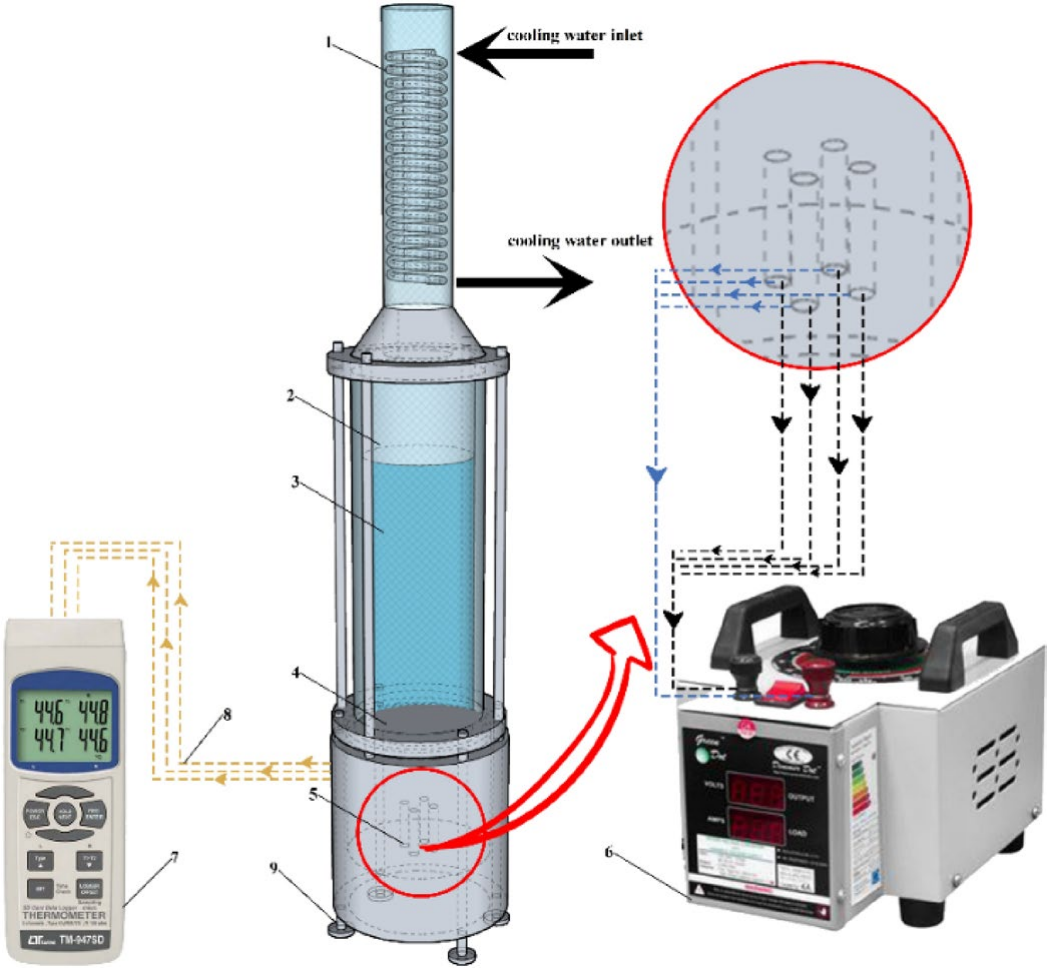
Schematic of the test apparatus. (1) Condenser (2) Pyrex glass (3) Boiling liquid (4) Test plate (5) Heater (6) Variac (7) Thermometer (8) Thermocouples (9) Retaining plate.

**Fig. 9:**
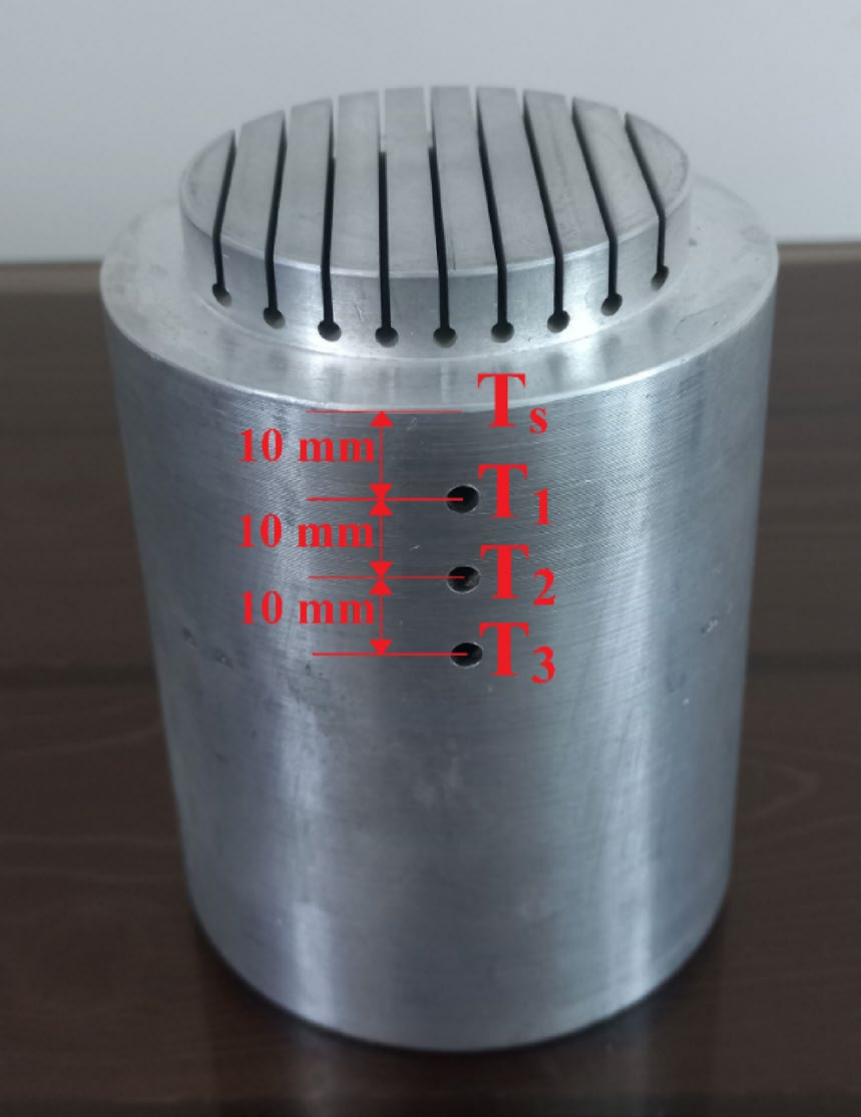
Image of the location of the thermal sensors in the grooved part under test.

**Fig. 10:**
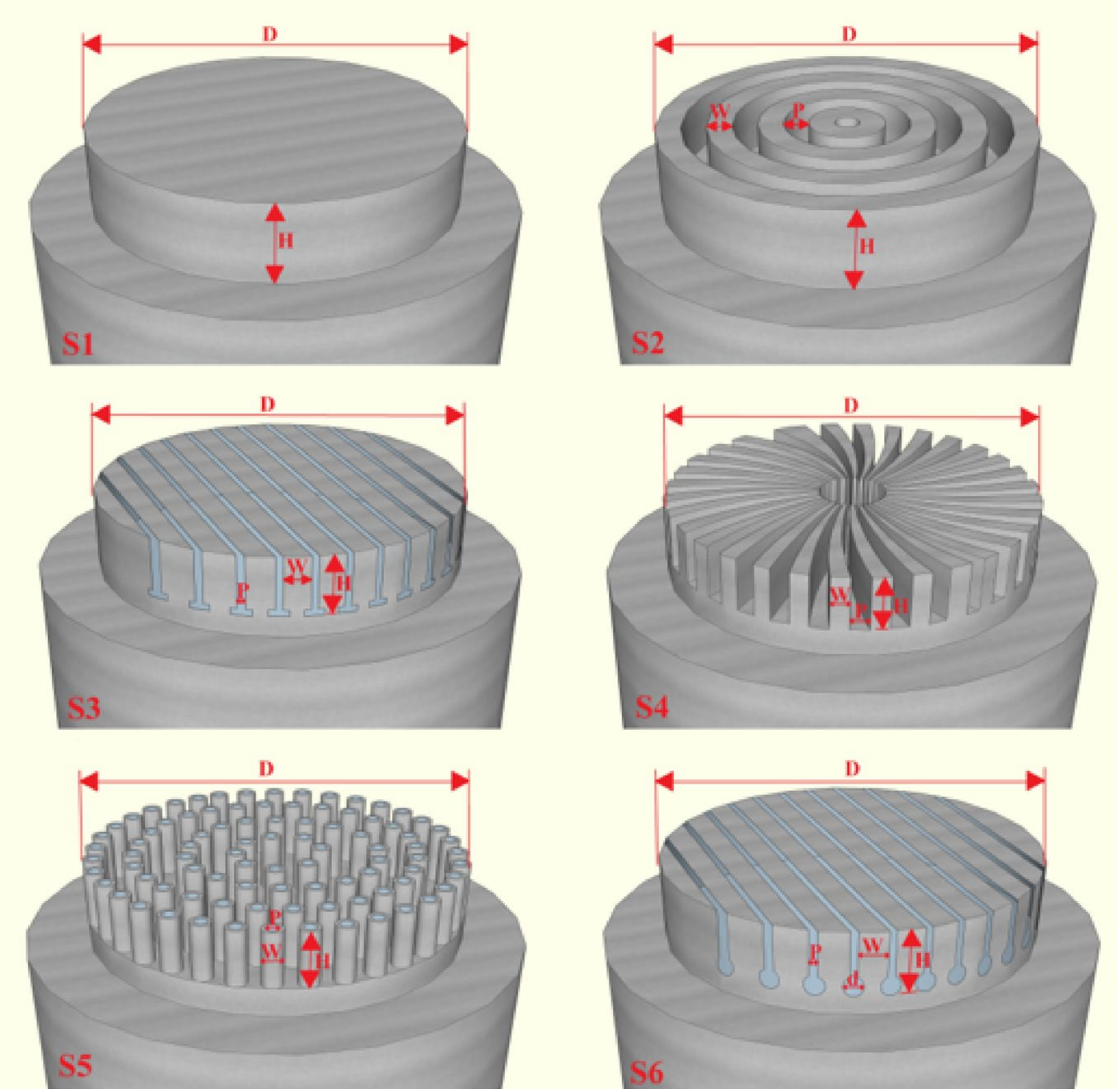
Schematic of fabricated grooved surfaces.

**Fig. 11:**
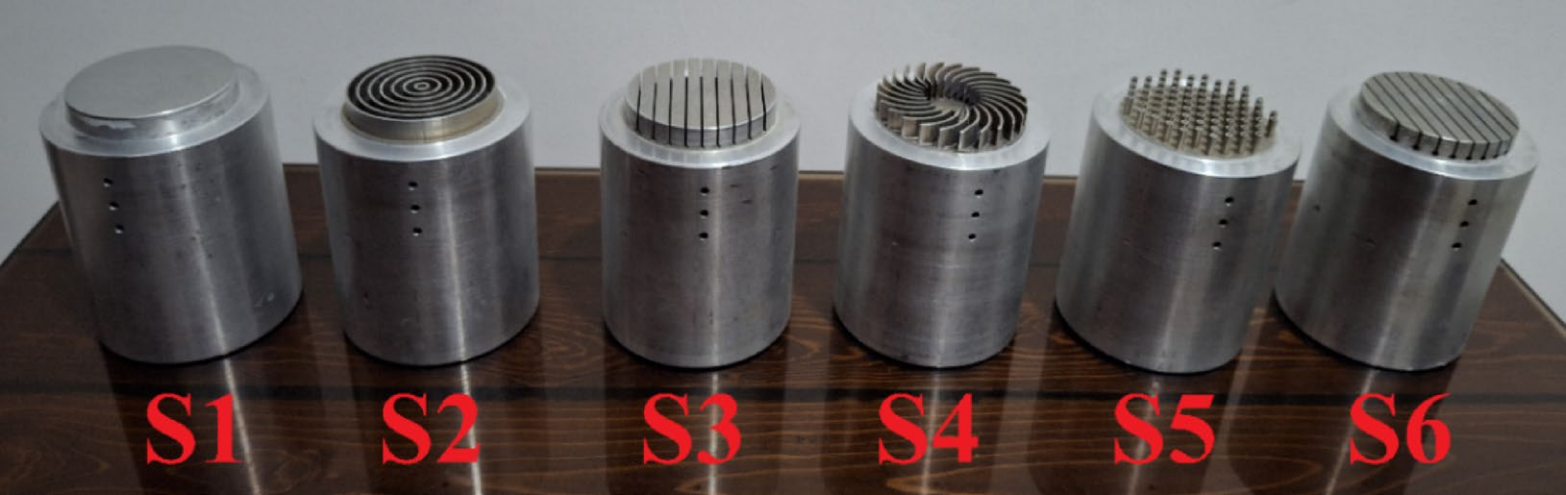
Actual images of fabricated grooved surfaces.

The aluminum heater and insulation assembly, the boiling tank, the condenser and cooling system, and the thermocouples are the major parts of the test setup, as illustrated in Figs. 7 and 8. The aluminum heater is made from the 7000 series aluminum alloy family and has a conductivity coefficient of 140 Wm^−1^ K^−1^ (Fig. 9). The aluminum heater is a cylindrical component of 80 mm diameter and 94 mm long. The heater surface is grooved, as shown in Figs. 10 and 11. Four holes, each with a diameter of 10 mm and a depth of 52 mm are drilled into the bottom of the aluminum heater to accommodate the cartridge elements. The heating elements are 250 watts in power, 10 mm in diameter, and 50 mm in length. Three holes are also made at specific distances from the heater surface to place type K thermocouples with an accuracy of ± 0.1 °C, as shown in Fig. 9. The insulator is a cylinder made of polytetrafluoroethylene with an inner diameter of 80 mm and an outer diameter of 150 mm. The thermal conductivity of both heater and polytetrafluoroethylene was measured by KD2 and reported to be 140 and 0.2 Wm^−1^ K^−1^, respectively. Due to the insulation’s low thermal conductivity, radial heat transfer can be disregarded, enabling the heat transfer to the heater surface to be treated as one-dimensional. The boiling tank is constructed from a Pyrex cylinder with the height, outer, and inner diameters of 400 mm, 74 mm, and 65 mm, respectively. The tank is constrained by two steel flanges positioned at its top and bottom. A condenser is employed to convert the boiling vapors back into liquid (the condensation process) and preserve the fluid volume. This Pyrex condenser measures 35 mm in diameter and 390 mm in length, with an open top since the experiments occur at atmospheric pressure. Water is pumped from a water/ice tank and channeled into the condenser to cool the circulating fluid, with the tank’s temperature maintained at 20 °C using a mercury thermometer.

### Boiling surfaces

**Fig. 12:**
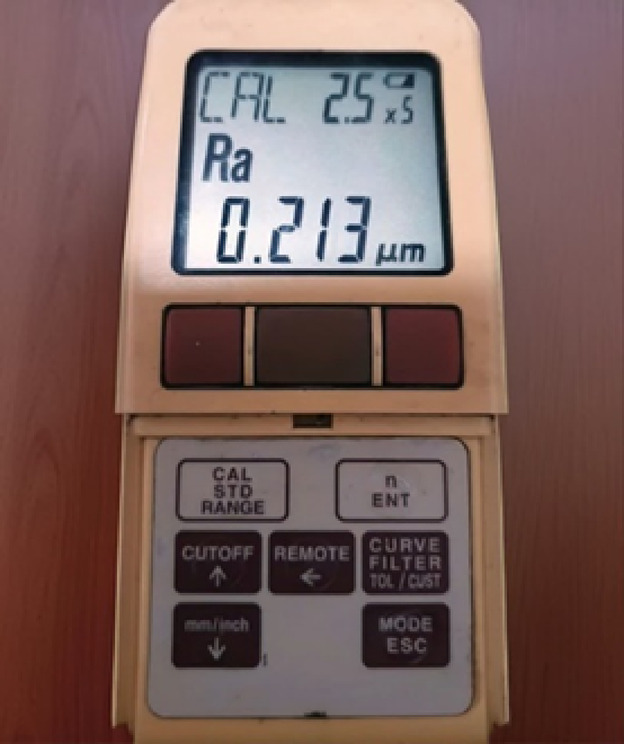
Mitutoyo surftest SJ-201 roughness meter.

The boiling surface’s diameter is 60 mm, and CNC and wire cutting groove it. The surface area of each is given in Table 2, and the dimensional specifications of the grooves are presented in Table 3. The roughness of the grooved surfaces was measured by a roughness meter(Mitutoyo, surftest:SJ-201) as shown in Fig. 12. The roughness of the boiling surfaces is 0.213 μm, on average. Given the average diameter of graphene oxide nanoparticles (3.4–7 nm) and iron oxide nanoparticles (20–30 nm), the parameter ⱷ, defined as the interaction between the surface and nanoparticles, exceeds a value of one.

**Table 2:** Areas of surfaces used in the experiment.

No:	Surface area (mm^2^)
S1	2826
S2	2826 + 12,874
S3	2826 + 7974
S4	2826 + 10,368
S5	2826 + 6697
S6	2826 + 8974

**Table 3:** Dimensional specifications of the grooves.

	S1	S2	S3	S4	S5	S6
D	60 mm	60 mm	60 mm	60 mm	60 mm	60 mm
W	**–**	1 mm	5 mm	1 mm	2 mm	5 mm
H	10 mm	10 mm	9 mm	9 mm	9 mm	9 mm
d	**–**	**–**	**–**	**–**	**–**	2 mm
P	**–**	1 mm	1 mm	4 mm	1 mm	1 mm

## Relationships

After obtaining the test results, it is necessary to use relationships to calculate the heat transfer coefficient (h). Due to the low thermal conductivity of PTFE, the high length-to-diameter ratio of the heater, the linear temperature profile along the heater length at a constant radius, and the nearly uniform temperature observed at different radii over the same heater length, radial heat transfer can be disregarded, enabling the heat transfer to the heater surface to be treated as one-dimensional. Therefore, for obtaining and estimating the surface temperature, assuming one-dimensional heat transfer, Eq. (3) can be applied to obtain the heat flux:

3 $${\text{q}}^{\prime \prime } = {\text{k}}\frac{{\partial {\text{T}}}}{{\partial {\text{x}}}} = {\text{k}}\frac{{{\text{T}}_{3} - {\text{T}}_{1} }}{{\text{x}}}$$

In the above equation, the values are x = 20 mm and k = 140 Wm^−1^ K^−1^, where x is the distance between thermocouples 1 and 3, k is the thermal conductivity of the heater, and T_1_ and T_3_ represent the temperatures measured by thermocouples 1 and 3 from the heater.

**Fig. 13:**
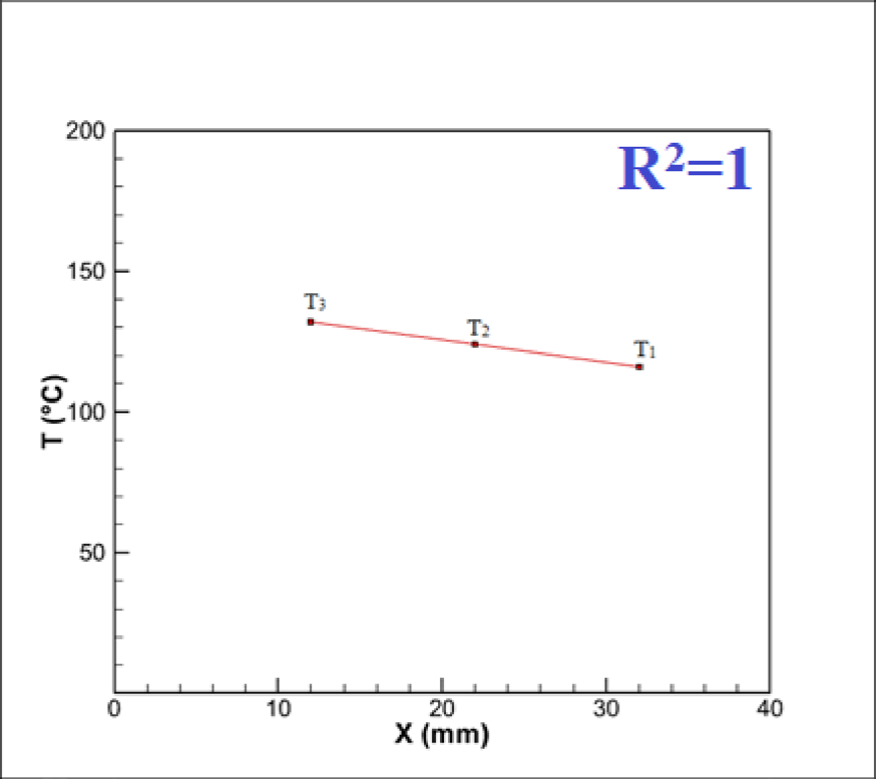
Temperature changes in the grooved block.

It is noteworthy that the temperature changes in the block are linear, and Fig. 13 (investigated in the laboratory) serves as proof of this claim:

Now, given the heat flux value q″, the surface temperature is obtained as below (4):

4 $${\text{T}}_{{\text{s}}} = {\text{T}}_{1} - {\text{q}}^{\prime \prime } \left( {\frac{{\text{L}}}{{\text{k}}}} \right)$$

For all grooved surfaces, L = 10 mm, which is equal to the distance between thermocouple 1 and the boiling surface and T_1 ,_ and T_1_ represent the temperature measured by thermocouple 1 from the heater.

On the contrary, we know that the excess temperature value ∆T can be obtained from Eq. (5):

5 $$\Delta {\text{T}} = {\text{T}}_{{\text{s}}} {-}{\text{T}}_{{{\text{sat}}}}$$

where T_sat_ is the saturated fluid temperature, and T_s_ is the grooved surface temperature.

Finally, the heat transfer coefficient can be obtained using Eq. (6):

6 $${\text{h}} = \frac{{{\text{q}}^{\prime \prime } }}{\Delta T}$$

### Uncertainty analysis

Two types of errors result in the deviation of experimental results from the actual ones: measurement errors and the human error. Measurement is often a result of miscalibration of the measuring equipment. This study utilizes the Holman method^37^ for analyzing uncertainty in experimental data. Here, U implies the measurement error. Thus, the uncertainties associated with heat flux, the temperature differential between the surface and the fluid, and the boiling heat transfer coefficient can be determined using Eqs. (7), (8), and (9), respectively. The measurement errors for temperature, distances, and thermal conductivity are recorded as 0.1 °C, 0.02 mm, and 0.038 Wm^−1^ K^−1^, respectively. Across all experiments, the maximum uncertainties observed were 6.15% for heat flux, 6.86% for the temperature differential between the surface and the fluid, and 6.61% for the boiling heat transfer coefficient.

7 $$\frac{{{\text{U}}_{{{\text{q}}^{\prime \prime } }} }}{{{\text{q}}^{\prime \prime } }} = \left( {\left( {\frac{{{\text{U}}_{{{\text{T}}3 - {\text{T}}1}} }}{{{\text{T}}_{3} - {\text{T}}_{1} }}} \right)^{2} + \left( {\frac{{{\text{U}}_{{{\text{X}}3 - {\text{X}}1}} }}{{{\text{X}}_{3} - {\text{X}}_{1} }}} \right)^{2} + \left( {\frac{{{\text{U}}_{{\text{k}}} }}{{\text{k}}}} \right)^{2} } \right)^{0.5}$$

8 $$\frac{{{\text{U}}_{{\Delta {\text{T}}}} }}{{\Delta {\text{T}}}} = \left( {\left( {\frac{{{\text{U}}_{{\text{L}}} }}{{\text{L}}}} \right)^{2} + \left( {\frac{{{\text{U}}_{{{\text{T}}3 - {\text{T}}1}} }}{{{\text{T}}_{3} - {\text{T}}_{1} }}} \right)^{2} + \left( {\frac{{{\text{U}}_{{{\text{X}}3 - {\text{X}}1}} }}{{{\text{X}}_{3} - {\text{X}}_{1} }}} \right)^{2} } \right)^{0.5}$$

9 $$\frac{{{\text{U}}_{{\text{h}}} }}{{\text{h}}} = \left( {\left( {\frac{{{\text{U}}_{{{\text{q}}^{\prime \prime } }} }}{{{\text{q}}^{\prime \prime } }}} \right)^{2} + \left( {\frac{{{\text{U}}_{{\Delta {\text{T}}}} }}{{\Delta {\text{T}}}}} \right)^{2} } \right)^{0.5}$$

## Results and discussion

**Fig. 14:**
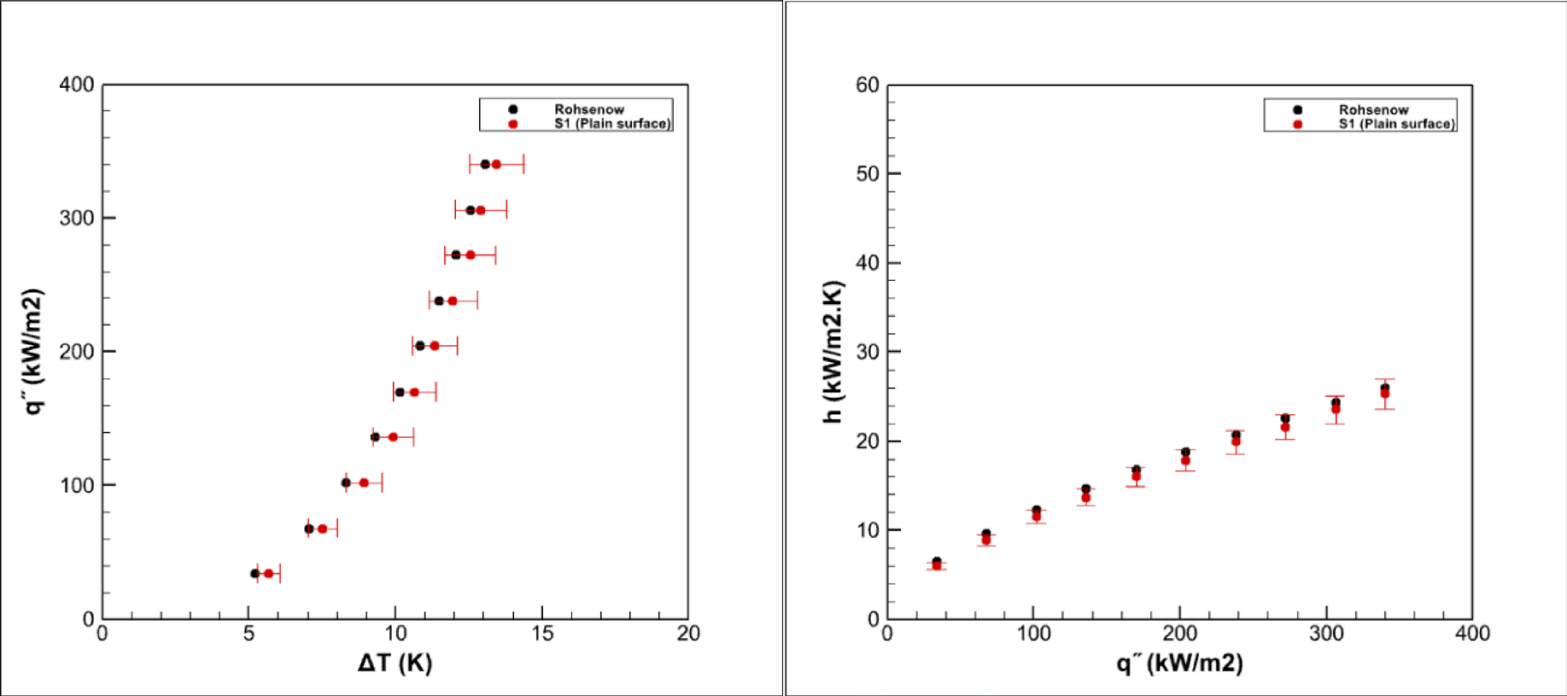
Comparison of the boiling curve of deionized water and the Rohsenow curve.

To ensure the reliability of the measurements, all experiments were repeated three times on different days under similar conditions. For validation purposes, pool boiling tests with deionized water were conducted on the smooth S1 surface, and the results were compared against the well-established Rohsenow correlation^38^. As illustrated in Fig. 14, the experimental boiling curve aligns closely with the theoretical prediction (average deviation ≈ 3.78% over the investigated heat flux range), thereby confirming the accuracy of the experimental setup and minimizing the possibility of systematic errors.

In the plot of heat transfer coefficient versus heat flux, a monotonic increase is observed, which is consistent with the fundamental mechanisms of nucleate boiling. At higher heat fluxes, the number of active nucleation sites increases and bubble formation becomes more intense, thereby enhancing convective heat transfer and leading to higher values of the heat transfer coefficient. Similarly, in the boiling curve presented as heat flux against wall superheat, the steep rise at larger temperature differences clearly reflects the transition into the fully developed nucleate boiling regime. The strong agreement between experimental data and the Rohsenow model not only validates the measurement procedure but also provides a solid reference baseline for evaluating the influence of hybrid nanofluids and surface modifications in the subsequent sections.

### Hybrid nanofluid boiling on grooved surfaces

**Fig. 15:**
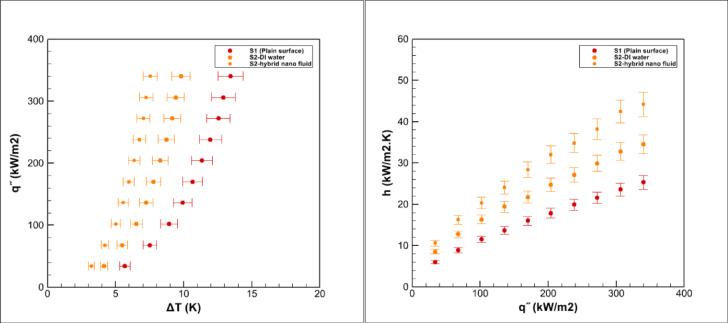
Heat transfer results in pool boiling for surface S2.

**Fig. 16:**
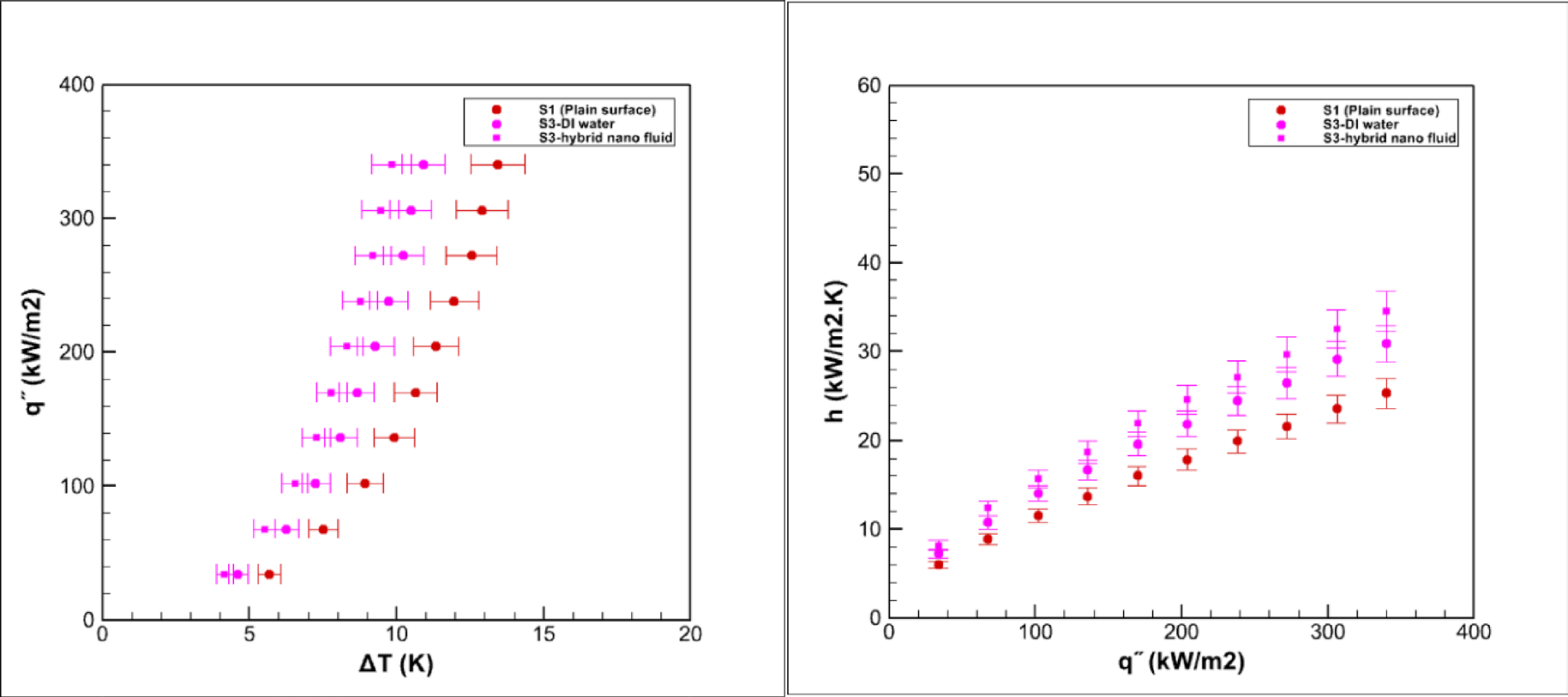
Heat transfer results in pool boiling for surface S3.

**Fig. 17:**
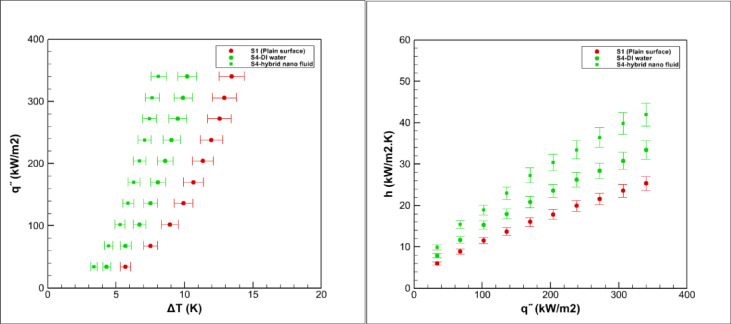
Heat transfer results in pool boiling for surface S4.

**Fig. 18:**
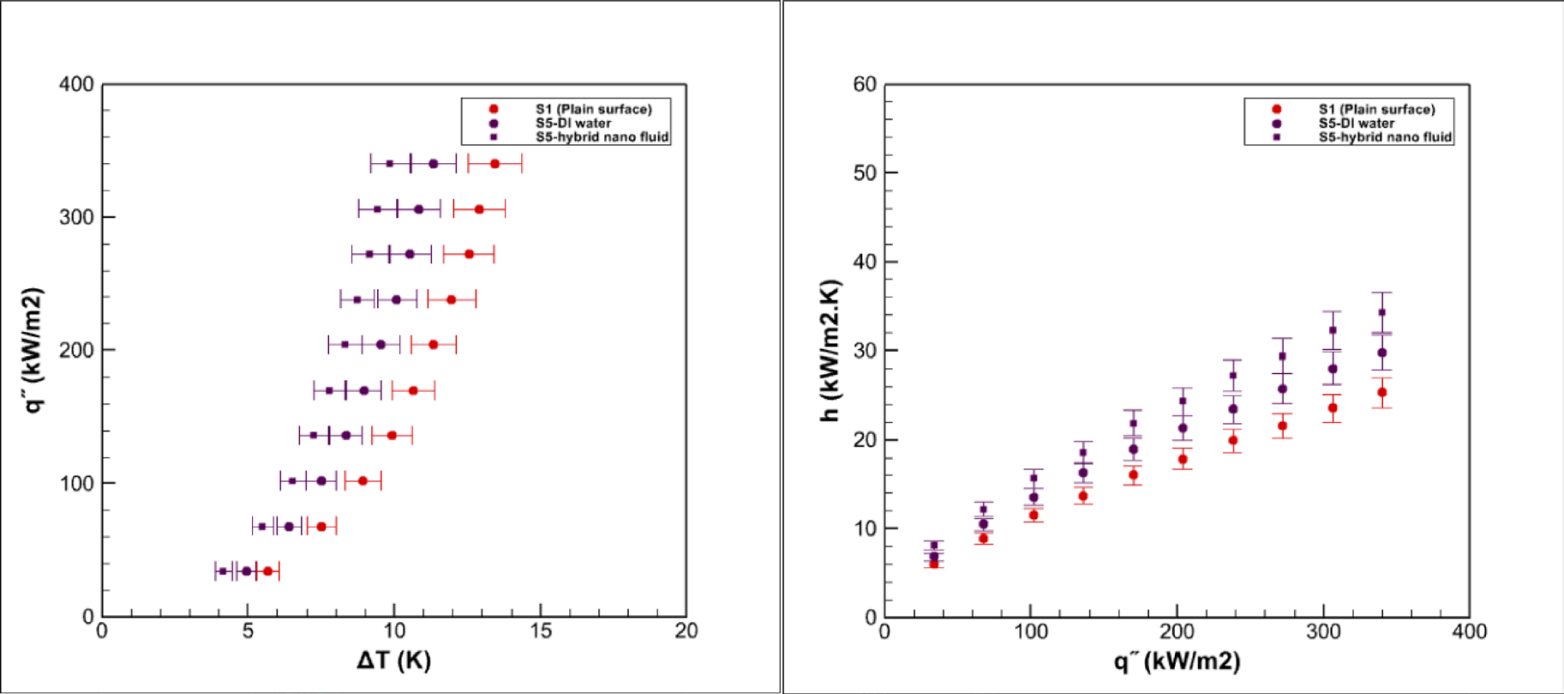
Heat transfer results in pool boiling for surface S5.

**Fig. 19:**
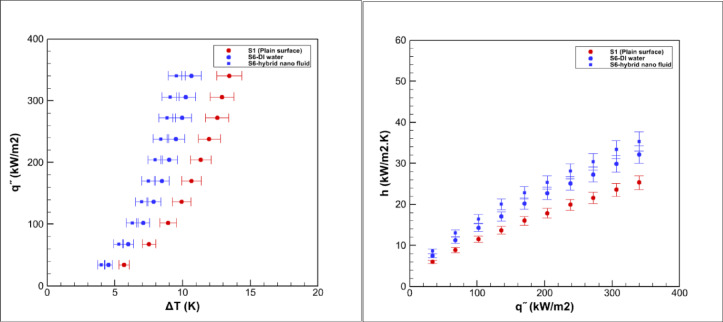
Heat transfer results in pool boiling for surface S6.

Pool boiling experiments were carried out on grooved surfaces using a hybrid nanofluid composed of graphene oxide and iron oxide nanoparticles dispersed in water at a volumetric concentration of 0.05%. The measured saturation temperature of this hybrid nanofluid was 98.9 °C, which was slightly higher than that of deionized water 96.7 °C, consistent with earlier reports for nanoparticle suspensions^3,5^. The boiling performance of the grooved surfaces is presented in Figs. 15, 16, 17, 18 and 19.

The results revealed a consistent leftward shift of the boiling curves for all surfaces tested with the hybrid nanofluid. This behavior indicates that the onset of nucleate boiling occurs at a lower wall superheat compared with pure water, in line with previous studies on nanofluids and hybrid nanofluids^5,14,15^. In other words, for the same heat flux, the required excess temperature difference is reduced. Such a trend clearly demonstrates the improvement in boiling efficiency provided by the hybrid nanofluid^14,15^.

Several mechanisms can explain this improvement. The inherently high thermal conductivity of graphene oxide and iron oxide nanoparticles enhances heat transport from the heated surface to the liquid^2,8^. In addition, the suspension of nanoparticles increases the effective heat transfer area and alters surface wettability, both of which encourage bubble nucleation^7,24^. The good stability of the nanofluid at the tested concentration ensures uniform dispersion of nanoparticles, while the combined effects of particle size, morphology, and hybrid composition further strengthen thermal conductivity and surface interactions^8,14^.

Moreover, since the enhancement factor (φ) was greater than unity, the number of active nucleation sites increased significantly, resulting in improved bubble dynamics and more efficient heat transfer^24^. Consequently, the overall heat transfer coefficient of grooved surfaces in contact with the hybrid nanofluid was consistently higher than that of the smooth reference surface^33^. Nonetheless, differences among various groove geometries were still evident and will be discussed in detail in the subsequent sections.

### Boiling on the S2 surface

Figure 15 illustrates the boiling performance on the S2 surface. Compared with the smooth reference surface (S1), the heat transfer coefficient on S2 increases by about 37% for deionized water and by nearly 67% for the hybrid nanofluid. The heat transfer coefficient (h) shows a continuous rise with increasing heat flux (q″), while the boiling curve (q″–ΔT) shifts to the left, indicating that nucleate boiling initiates at a lower wall superheat.

The notable enhancement observed on S2 can be attributed to its specific geometry, consisting of concentric circular grooves extending across the surface. This arrangement enlarges the effective heat transfer area and provides a high density of nucleation sites, which promotes frequent bubble generation. The symmetric groove pattern also distributes vapor bubbles uniformly and guides their radial movement toward the edges, thereby accelerating bubble departure and preventing vapor accumulation in the central region. Furthermore, the groove structure facilitates rapid rewetting of the surface by channeling liquid back into the cavities, which delays local dry-out and sustains efficient boiling.

When combined with the superior thermal conductivity of the graphene oxide–iron oxide hybrid nanofluid, these geometric advantages lead to intensified bubble dynamics, stronger liquid agitation, and higher overall heat transfer. This synergy explains the substantial performance improvement of the S2 surface compared with both the smooth reference and other tested geometries.

### Boiling on the S3 surface

Figure 16 presents the boiling performance on the S3 surface. Compared with the smooth reference, the average heat transfer coefficient increases by about 23% for deionized water boiling and by 35% for hybrid nanofluid boiling. Although these results confirm a clear improvement, the overall enhancement is less pronounced than that observed for the S2 surface.

This behavior is primarily influenced by the unique geometry of S3, which consists of parallel grooves with relatively deep and partially vertical sections. While this design enlarges the effective surface area and provides additional nucleation sites, the vertical walls reduce surface wettability and create larger bubble contact angles compared with horizontal surfaces. As a result, bubbles tend to adhere longer before detachment, delaying liquid replenishment at the heated wall and limiting the intensity of convective mixing.

When boiling the hybrid nanofluid, the intrinsic thermal conductivity of the suspended nanoparticles still contributes to performance gains, yet the geometric constraints of S3 hinder the full utilization of these advantages. Consequently, although the S3 surface exhibits higher heat transfer performance than the smooth baseline, its efficiency remains below that of the more favorable S2 configuration.

### Boiling on the S4 surface

Figure 17 illustrates the boiling performance of the S4 surface, showing a marked enhancement in heat transfer. Compared with the smooth reference, the average heat transfer coefficient increases by ~ 32% for deionized water boiling and ~ 60% for hybrid nanofluid boiling. The (h–q″) relationship exhibits a steeper rise than that of the plain surface, while the boiling curve (q″–ΔT) shifts distinctly to the left, indicating the onset of nucleate boiling at a lower wall superheat.

This improvement is attributed to the radial fin geometry of S4. The enlarged surface area introduces numerous nucleation sites, and the uniform fin spacing promotes orderly bubble departure while suppressing vapor entrapment. The absence of sharp corners enables smooth bubble detachment and rapid migration through the channels, thereby reducing wall thermal resistance. In addition, the radial layout directs vapor outward from the center, ensuring effective vapor removal and continuous liquid replenishment in the grooves.

When combined with the high thermal conductivity of the hybrid nanofluid and its favorable effect on surface wettability, these geometric features foster vigorous bubble dynamics and intense convective mixing near the heated wall. Consequently, the S4 surface demonstrates one of the most significant enhancements in boiling heat transfer among the tested configurations.

### Boiling on the S5 surface

Figure 18 reports the boiling performance of the S5 surface. Relative to the smooth reference, the average heat transfer coefficient increases by about 19% with deionized water and by 34% with the hybrid nanofluid. The (h–q″) trend rises more steeply than that of the plain surface, and the (q″–ΔT) curve exhibits a leftward shift, though the shift is less pronounced than for the most effective geometries (e.g., S2 and S4).

The behavior of S5 is governed by its array of thin-walled hollow cylinders arranged over the heating area. This topology enlarges the effective area by providing both inner and outer cylindrical surfaces and creates a high density of active nucleation perimeters along the tube rims, which promotes frequent bubble inception and departure. Capillary wicking inside the tubes helps replenish liquid to the hot wall and delays local dry-out.

At the same time, the confined bores can stabilize vapor pockets—especially when the depth-to-diameter ratio is high—so bubbles may linger inside the tubes before venting. The resulting reduction in liquid exchange within some cavities limits local convective mixing and partially offsets the geometric benefits. Consequently, S5 outperforms the smooth baseline but delivers a smaller overall enhancement than S2 and S4. The hybrid nanofluid’s superior effective thermal conductivity and wettability further assist bubble dynamics on S5, yet the confinement inherent to the hollow-tube geometry caps the attainable gains.

### Boiling on the S6 surface

Figure 19 illustrates the boiling behavior on the S6 surface. Relative to the smooth baseline, the average heat transfer coefficient increases by 26% during deionized water boiling and by 39% with the hybrid nanofluid. Although the enhancement is clear, it remains below that observed for the most efficient geometries such as S2 and S4.

The performance of S6 is dictated by its linear grooves terminated with circular cavities. This configuration enlarges the effective heat transfer area and generates numerous potential nucleation sites, particularly along the rim of the cavities. The terminal cavities also serve as micro-reservoirs that retain liquid and facilitate rapid surface rewetting, which helps delay local dry-out.

However, the same cavities can also act as traps for vapor bubbles. When bubbles accumulate inside these recessed regions, they tend to linger longer before departure, which reduces the rate of vapor release and promotes the formation of a thin vapor layer. This effect limits convective mixing and partially offsets the geometric benefits. As a result, while S6 significantly outperforms the plain surface and shows stronger gains when combined with the hybrid nanofluid, its overall efficiency remains lower than that of S2 and S4.

It is important to note that, although the observed boiling enhancements are partly attributed to changes in surface wettability, direct contact-angle measurements were not performed in this work. Therefore, the discussion of wettability effects is qualitative and based on established findings from the literature. Previous studies^7,14,15,21,24^ have shown that the addition of nanoparticles can reduce the contact angle and improve surface rewetting, which promotes bubble nucleation and enhances HTC. While the present results are consistent with these trends, a detailed quantitative assessment of wettability is left for future investigations.

**Fig. 20:**
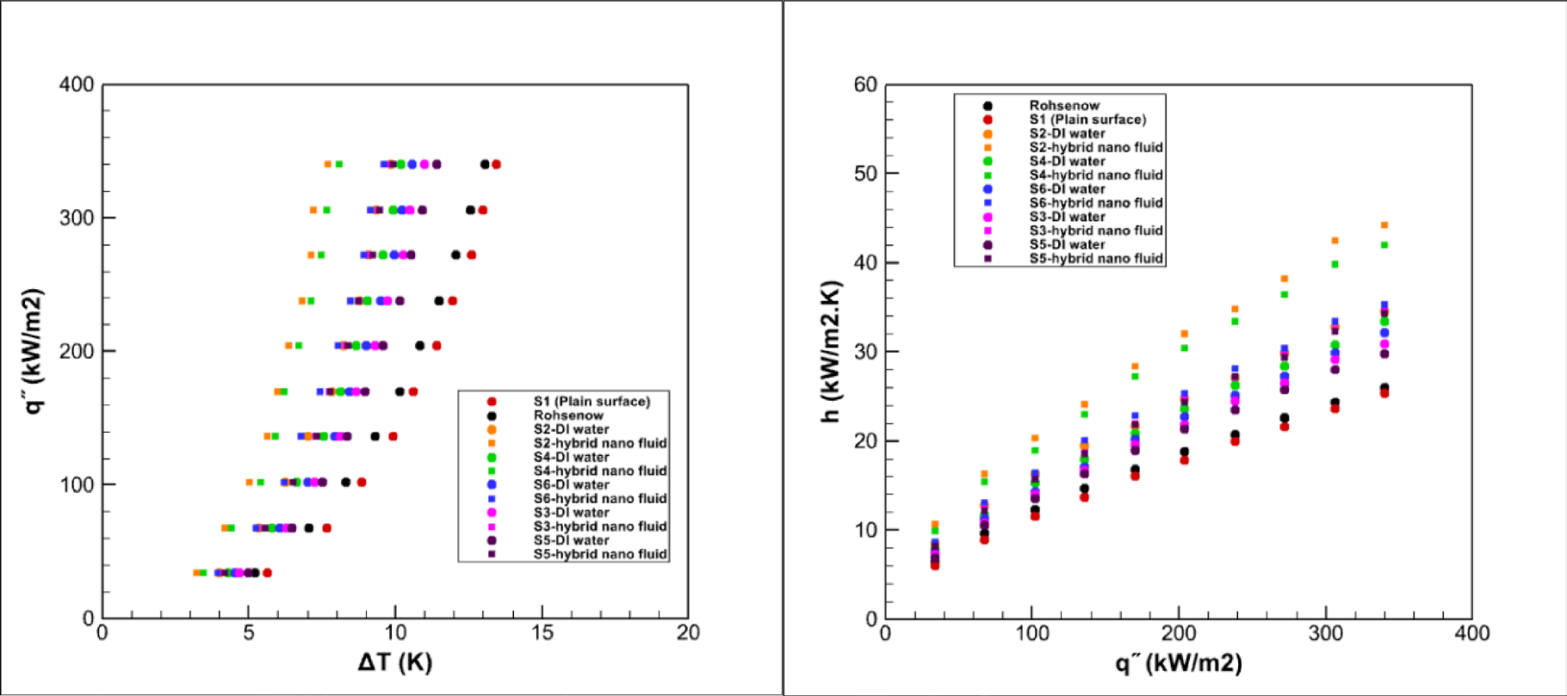
Heat transfer results in pool boiling for all surfaces.

For a clearer comparison among all tested surfaces, the overall heat transfer results in pool boiling are presented in Fig. 20. This figure illustrates the variation of the heat transfer coefficient with heat flux for each surface and allows direct evaluation of their relative performance.

## Conclusion

In the present work, the GO–Fe_3_O_4_/water hybrid nanofluid exhibited a considerable improvement in the heat transfer coefficient (HTC) compared with deionized water, particularly on the grooved S2 surface. The obtained enhancements are positioned toward the higher end of the values reported for hybrid nanofluids in recent studies. For instance, Ma et al.^18^ reported notable boiling performance using graphene–silver hybrids, Kamel et al.^15^ observed about 37% HTC enhancement with Al_2_O_3_/CeO_2_ nanofluids, and Mehralizadeh et al.^16^ achieved similar improvements for TiO_2_/SiO_2_ hybrids on modified surfaces. The relatively stronger and more consistent performance observed in the present study can be attributed to the synergistic influence of the GO–Fe_3_O_4_ hybrid composition and the optimized grooved surface geometry, which together enhanced surface–liquid interactions and bubble dynamics. Therefore, the current findings not only align well with the literature but also extend it by demonstrating a combined fluid–surface approach for achieving more stable and efficient nucleate pool boiling heat transfer.

**Fig. 21:**
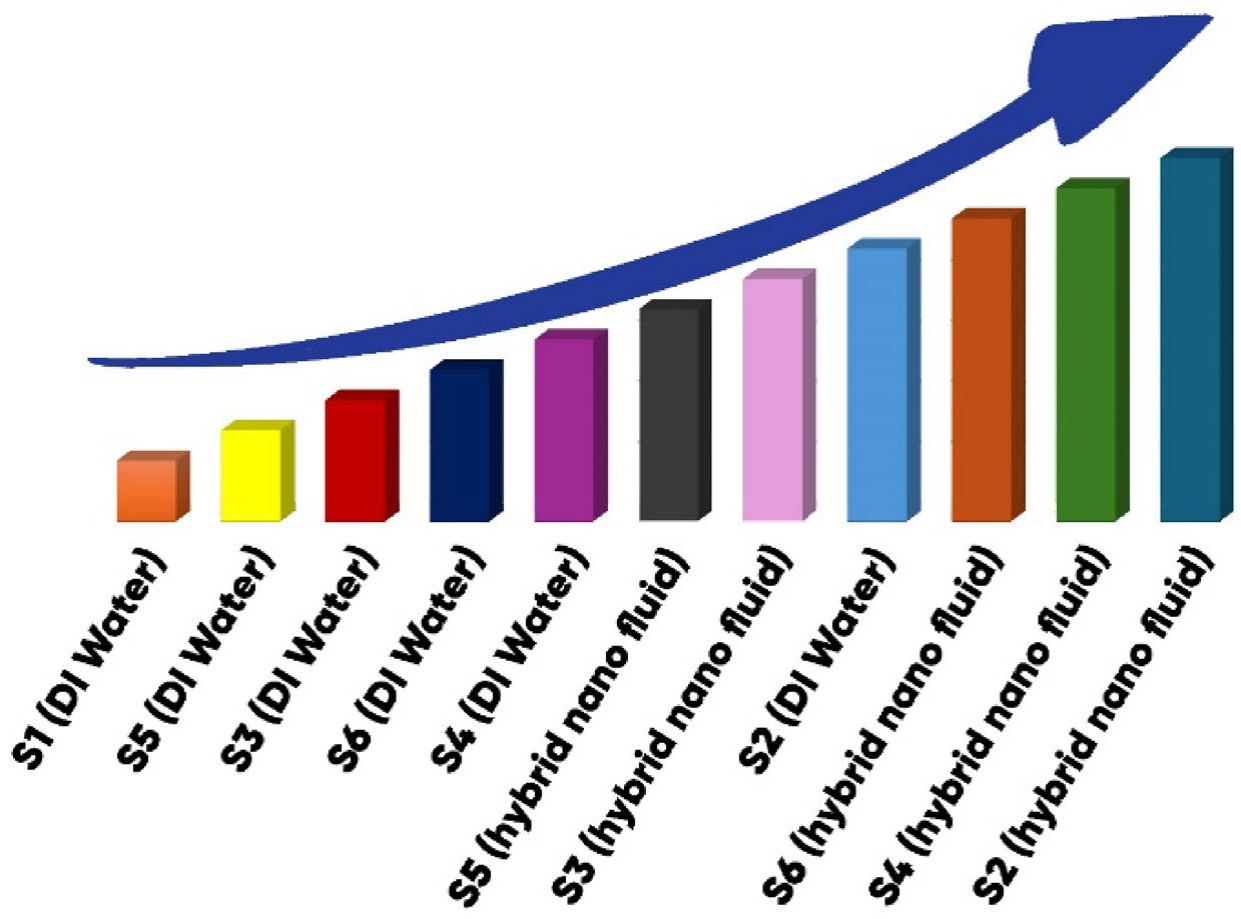
The overall trend of heat transfer enhancement in pool boiling.

This study conducts an experimental analysis of the heat transfer coefficient in pool boiling, employing a hybrid nanofluid composed of graphene oxide, iron oxide, and water (at a 0.05% volumetric concentration) on different grooved surfaces. The primary results are outlined as follows:

1. The hybrid nanofluid significantly enhanced heat transfer compared to deionized water, owing to its higher thermal conductivity and improved nucleation characteristics.
2. The S2 surface delivered the highest heat transfer coefficient and heat flux rate when boiling the hybrid nanofluid.
3. Despite geometric drawbacks, surfaces with larger effective area (e.g., S3 and S6) demonstrated improved heat transfer relative to S1.
4. The hybrid nanofluid demonstrated excellent stability, which contributed to the improvement of the overall system performance.
5. Overall, the graphene oxide–iron oxide hybrid nanofluid, combined with grooved surfaces, offers a promising approach for enhancing pool boiling heat transfer.
6. It was observed that surfaces S5 and S3 showed poor heat transfer enhancements during the deionized water boiling. However, they demonstrated significantly improved performance in hybrid nanofluid boiling, outperforming surfaces S4 and S6 in deionized water boiling.
7. Finally, the overall trend of heat transfer improvement observed throughout the experiments is depicted in Fig. 21.

These findings highlight the potential application of hybrid nanofluids with engineered surfaces in high-heat-flux thermal management systems such as electronics cooling and nuclear reactors. Despite these promising results, the surface wettability was not directly measured; therefore, related effects were inferred from the literature. Furthermore, this work was confined to the nucleate boiling regime with emphasis on the heat transfer coefficient (HTC), while critical heat flux (CHF) was beyond the scope of this study. Future research should address these aspects through durability testing, direct wettability characterization, optimization of nanoparticle combinations, and broader operating conditions to further assess the industrial applicability of this approach.

## Data Availability

The data that support the ﬁndings of this study are available from the corresponding author upon reasonable request.

## List of symbols

L: Distance between the thermocouple and surface mm
h: Convection heat transfer coefficient Wm^−^^2^ K^−^^1^
q˝: Heat flux Wm^−^^2^
T: Temperature K
k: Thermal conductivity Wm^−^^1^ K^−^^1^
x: Position of thermocouple mm
ΔT: Temperature difference K
U: Uncertainty
V: Volume m^3^

## Greek symbols

Δ: Difference
ϕ: Concentration by volume %
ρ: Density kg m^−3^
μ: Viscosity mPa s

## Subscripts

s: Surface
sat: Saturation
bf: Base fluid
hnf: Hybrid nanofluid
np: Nano particles

## Abbreviations

Fe_3_O_4_: Iron oxide
GO: Graphene oxide
SEM: Scanning electron microscope
TEM: Transmission electron microscopy
HTC: Heat transfer coefficient
CHF: Critical heat flux
